# Supplementary material for: Biodiversity–production feedback effects lead to intensification traps in agricultural landscapes
Source: Nat Ecol Evol. 2024 Mar 6;8(4):752–60. doi: 10.1038/s41559-024-02349-0 (PMC11009109; doi:10.1038/s41559-024-02349-0)
Supplement: Supplementary file 1 — Sections A1–A5, Supplementary Figs. 1–4 and Supplementary Tables 1–4. A1: Detailed description of model structure, A2: Model parametrisation, A3: Simplifications in model construction, A4: Additional tables and figures, A5: References. [file 41559_2024_2349_MOESM1_ESM.pdf]

nature ecology & evolution

Article https://doi.org/10.1038/s41559-024-02349-0

Biodiversity–production feedback effects lead to intensification traps in agricultural landscapes

In the format provided by the authors and unedited

Table of content:

A1: Detailed description of model structure
A2: Model parametrisation
A3: Simplifications in model construction
A4: Additional tables and figures
A5: References

A1: Detailed description of model structure

General summary

An overview of the model structure is provided in Fig. 1 and this detailed description of the model's formulation will follow the presentation of key relationships outlined in the figure. The aim of our model was to assess how different land-use scenarios interact with landscape characteristics in their influence on total production (P_T) and total biodiversity (B_T) in a landscape. Land management was defined as (i) the fraction of land used for agricultural production in a landscape (i.e. working land, WL) and (ii) the external input effort such as fertiliser and pesticide inputs, invested in agricultural production per area (i.e. intensification effort, I_E). In our simulations, we only assessed conventional intensification and did not consider options of ecological intensification. Ecological intensification can reach similar or sometimes even higher levels of productivity (e.g. Pywell *et al.* 2015), but is also frequently related to substantially higher working costs. The integration of working costs and parametrisation of the relationship between ecological intensification and working costs is not trivial and was beyond the scope of this study. I_E and WL are also referred to as input variables as they can be determined by land managers.

In our model, crop, soil and biotic characteristics of a landscape are influencing the form of five key relationships (Fig. 1) which together define the response of P_T and B_T to input variables. In Supplementary Table 1, the variables which influence these five relationships are summarised, which are further referred to as model constants. Finally, P_T, B_T and the average yield of cultivated land (defined here as production per area, Y) are computed based on the input variables and model constants and are hence referred to as output variables.

The final mathematical output of the model is P_T which was computed based on the formula:

$$P_T = WL\ Y \quad (1)$$

However, P_T does not only depend directly on WL and Y but also on a number of first order and second order indirect relationships, which are highlighted in Fig. 1. First order indirect relationships can be summarised by the formula

$$Y = Y_{Max}\, f_{I_E}^{Y}(I_E)\ f_{B_T}^{Y}(B_T)\, f_{WL}^{Y}(WL) \quad (2)$$

where Y_{Max} stands for the maximum achievable yield, while $f_{I_E}^{Y}(\cdot), f_{B_T}^{Y}(\cdot)$, and $f_{WL}^{Y}(\cdot)$ are functions governing the impacts of intensification effort, total biodiversity, and fraction of land used for production on Y, respectively. These terms will be defined further below. Y_{Max} was for simplicity kept at 1 for all landscapes, which led to a normalisation of productivity differences across landscapes. Second order indirect relationships can be denoted as

$$B_T = f_{I_E\,WL}^{B}(I_E, WL) \quad (3)$$

Hence, Y and B_T can be derived from five pairs of predictor terms (for mathematical reasons, the two predictors affecting biodiversity are summarised in one function in Equation 3), which refer to the five key relationships in Fig. 1. Based on these relationships (detailed functions are explained below), we established for each landscape (i.e., one specific set of model constants) the response of output variables to different land management options (i.e. input variables). Different land management options were evaluated by creating a model grid that contained 10,201 different combinations of input variables. Specifically, both WL and I_E ranged from 0 to 100% and were scaled in 1% steps. This land-management matrix contained all possible combinations of WL and I_E (101 x 101 combinations). We further refer to the assessment of P_T, B_T and Y under one given land-use regime (one WL and I_E combination) in a specific landscape as one model scenario whereas the assessment of all possible land-use regimes in a landscape is referred to as one model run.

Specific description of functional relationships

In this section, we specify, the four functions listed in the equation (2) and (3) representing the five key relationship defined in Fig. 1. Equations 4-11 thereby specify the function in equation (3) and equations (12-16) the functions used in equation (2).

Relationships D (the effect of I_E on the realised biodiversity in a landscape) and relationship E (the effect on land-use on the potential biodiversity in a landscape) can be summarised as:

$$f^{B}_{I_E\,WL}(I_E, WL) = B^{WL}_{pot.}\ B^{WL}_{loss} + B^{nonWL}_{pot.}\ B^{nonWL}_{loss} \quad (4)$$

where $B^{WL}_{pot.}$ and $B^{nonWL}_{pot.}$ stand for the potential diversity in *WL* and *nonWL* (fraction of non-working land in a landscape) and $B^{WL}_{loss}$and B^{nonWL}_{loss} stand for the proportion of biodiversity remaining after accounting for the negative impact of I_E in *WL* and *nonWL*, respectively.

Relationship E

Relationship E describes the impact of land-use on the potential number of species that can persist in a landscape. In our model, this was implemented by first creating a regional pool of *n* species that could potentially colonise and co-exist in the landscape. *n* was set to 10 000 across all of our simulations. Each species in this species pool was characterised by two traits; its habitat requirements (H_{Ii}) and its minimum habitat threshold level of existence (H_{Ti}). H_{Ii} could be one of three possible categories including (a) species that could only reproduce in semi-natural and natural habitat, i.e. *nonWL*, (b) species that could only reproduce in *WL* and (c) species that could reproduce in both *WL* and *non-WL* (*both*). Attribution of species to the three possible categories was a stochastic process based on predefined probabilities. These predefined probabilities (i.e. the expected proportion of species, which are able to reproduce in *WL*, *non-WL* and in both habitat types) represents a landscape characteristic (model constant) as they depend on physiochemical conditions shaping community assembly processes (e.g. Kraft *et al.* 2015). However, we want to emphasise that we define habitat requirements explicitly as the habitat that is required for reproduction (e.g. dead wood, clay soils, etc.). Species that require a certain habitat type for reproduction are still expected to use other habitat types for resource allocation and hence a "natural-habitat species" can have an impact on crop production.

Further, each species in the species pool had a minimum habitat threshold level of existence (H_{Ti}), which represented the minimum fraction of suitable habitat (H_S) a species requires to survive (e.g. a H_T of 0.2 indicates that a population of a species needs 20% of suitable habitat

in a landscape to persist). The association of a H_T to each species was again a stochastic process based on a truncated normal distribution (i.e. only H_T values between 0 and 1 were possible), which was defined based on a mean and a standard deviation that was constant within one model scenario. Hence, the expected mean and standard deviation of H_T are both further landscape characteristic (model constants, see Supplementary Table 2).

Based on H_{Si} and H_{Ii} of *n* species, we compute the species richness that is maintained in *WL* ($Richness_{WL}$) and *nonWL* ($Richness_{nonWL}$) based on the formula:

$$Richness_{WL} = \sum_{i=1}^{n} \mathbf{1}(H_{Si} > H_{Ti}, H_{Ii} = WL) + WL\ 1(H_{Ii} = both) \quad (5)$$

$$Richness_{nonWL} = \sum_{i=1}^{n} \mathbf{1}(H_{Si} > H_{Ti}, H_{Ii} = nonWL) + nonWL\ 1(H_{Ii} = both) \quad (6)$$

where **1**(·) denotes an indicator function which returns the value 1 when the condition is fulfilled and the value 0 if it is not fulfilled. Note that the second term in equation (5) and (6) was included to allocate the species, which can reproduce in both *WL* and *nonWL* and hence always have 100 percent suitable habitat available, to *WL* and *nonWL* based on the relative ratio of the two habitat types in a model run. $Richness_{WL}$ and $Richness_{nonWL}$ were then summed to attain $Richness_{Total}$, the total potential species richness that can be attained in a landscape given a certain land-use pattern.

Finally, we calculated $B_{pot.}^{WL}$ and $B_{pot.}^{nonWL}$ for each model scenario *k* of the *t* total model scenarios in one run (i.e. 10,201) as

$$B_{pot.}^{maxTotal} = \max(f_u : u = Richness_{WL}^{1} + Richness_{nonWL}^{1}, \ldots, Richness_{WL}^{t} + Richness_{nonWL}^{t}) \quad (7)$$

$$B_{pot.}^{WL} = \frac{Richness_{WL}^{k}}{B_{pot.}^{maxTotal}} \quad (8)$$

$$B_{pot.}^{nonWL} = \frac{Richness_{nonWL}^{k}}{B_{pot.}^{maxTotal}} \quad (9)$$

which effectively standardised the $B_{pot.}^{WL}$ and $B_{pot.}^{WL}$ by $B_{pot.}^{maxTotal}$, the maximal potentially attainable total species richness in a landscape.

Relationship D

After establishing the potential biodiversity in different habitat types, we compute B_{loss}^{WL} and B_{loss}^{nonWL} which together determine the percentage of biodiversity (i.e. species), which is lost due to conventional intensification. Conventional intensification comprises different activities such as the use of herbicides and insecticides or fertilisers. Our basal assumption was that I_E has always a negative impact on B_T. However, the shape of this relationship and the maximal impact of I_E on biodiversity can be highly variable and depends e.g. on the stress resistance of species in the species pool (landscape characteristic, Fig. 1). In our model, the two model constants $Shape_{BI}$ and $Impact_{BI}$ regulate the shape of the relationship and the maximal effect size of I_E on B_T independently from each other. $Impact_{BI}$ can be a value between 0 (no impact) and 1 (i.e. when I_E is 1 B_T is 0). $Shape_{BI}$ values can fall in the range between -1 (strongly

convex relationship) and 1 (strongly concave relationships) with 0 representing a linear relationship (Supplementary Figure 1).

B^{WL}_{loss} and B^{nonWL}_{loss} are computed separately, because species in *WL* are directly affected by I_E whilst species in *nonWL* are only affected indirectly via negative spill-over effects ($Spill_{IE}$) caused e.g. by pesticide applications (Brühl *et al.* 2021). The decrease of $Spill_{IE}$ over space is accounted for by linking the constant to *WL*. B^{WL}_{loss} and B^{nonWL}_{loss} are hence computed as:

$$B^{WL}_{loss} = \begin{cases} (1 - I_E)^{\left(1+ Shape_{BI} + 6\, Shape_{BI}^{3}\right)}\, Impact_{BI} + \, (1 - Impact_{BI}) & if\ Shape_{BI} \geq 0 \\ 1 - I_E{}^{\left(1+ |Shape_{BI}| + 6\, |Shape_{BI}|^{3}\right)}\, Impact_{BI} & if\ Shape_{BI} < 0 \end{cases} \quad (10)$$

$$B^{nonWL}_{loss} = \begin{cases} (1 - I_E)^{\left(1+ Shape_{BI} + 6\, Shape_{BI}^{3}\right)}\, Impact_{BI}\, WL^{Spill_{IE}} & if\ Shape_{BI} \geq 0 \\ \qquad + (1 - Impact_{BI}\, WL^{Spill_{IE}}) & \\ 1 - I_E{}^{\left(1+ |Shape_{BI}| + 6\, |Shape_{BI}|^{3}\right)}\, Impact_{BI}\, WL^{Spill_{IE}} & if\ Shape_{BI} < 0 \end{cases} \quad (11)$$

Based on B^{WL}_{loss} and B^{nonWL}_{loss} as well as $B^{WL}_{pot.}$ and $B^{nonWL}_{pot.}$ (derived from equations 8 and 9), B_T can be computed after equation (4) for each scenario in a model run.

Relationship C

Relationship C describes the impact of B_T on average *Y* in an agricultural landscape and is represented by the function $f^{Y}_{B_T}(B_T)$ in equation 2. Analogously to the computation of LB_{WL}, we again establish an effect size ($Impact_{YB}$) and a shape parameter ($Shape_{YB}$). $Impact_{YB}$ is again scaled from 0 (no reduction in *Y* when B_T is 0) to 1 (*Y* is 0 when B_T is 0). $Shape_{YB}$ ranges from -1 (concave) to 1 (convex relationships; Supplementary Figure 1). $f^{Y}_{B_T}(B_T)$ is expressed as

$$f^{Y}_{B_T}(B_T) = \begin{cases} B_T{}^{\left(1+ Shape_{YB} + 6\, Shape_{YB}^{3}\right)}\, Impact_{YB} + \, (1 - Impact_{YB}) & if\ Shape_{YB} \geq 0 \\ 1 - (1 - B_T)^{\left(1+ |Shape_{YB}| + 6\, |Shape_{YB}|^{3}\right)}\, Impact_{YB} & if\ Shape_{YB} < 0 \end{cases} \quad (12)$$

Relationship B

Relationship B represents the impact of I_E on *Y* described by the function $f^{Y}_{I_E}(I_E)$ in equation 2. Again, the relationship is dependent on two constants representing effect size ($Impact_{YI}$) and relationship shape ($Shape_{YI}$). However, in contrast to relationships C and D, we only considered concave relationship shapes for the impact of I_E on *Y* ($-1 \leq Shape_{YI} \leq 0$) as this relationship type is generally conceptually assumed (e.g. Liu *et al.* 2018) and found in field investigations (Scaife 1968). $f^{Y}_{I_E}(I_E)$ is expressed as:

$$f^{Y}_{I_E}(I_E) = 1 - (1 - I_E)^{\left(1+ |Shape_{YI}| + 6\, |Shape_{YI}|^{3}\right)}\, Impact_{YI} \quad (13)$$

Relationship A

Finally, modelled landscapes are designed to show heterogeneity in their maximal potential productivity (i.e. not all areas are equally fertile). Areas attributed to *WL* are always implemented on the most productive land. E.g. a value of 0.2 for *WL* implies that the 20 most productive percent of the landscape are used for agricultural production. We describe the

heterogeneity of potential productivity in modelled landscapes based on a beta-distribution. Beta-distributions are naturally constraint to values ranging between 0 and 1 and the shape of distributions can be described by its mean and variance. However, in contrast to normal distributions, the variance of the beta-distribution is logically constrained to certain values, which results from the limited data-range of the distribution (i.e. if values are restricted to range from 0 to 1, variance cannot be 100). This restriction of the variance becomes stronger when mean values approach 0 or 1 (Supplementary Figure 2).

The mean ($Ypot_{mean}$) and the variance ($Ypot_{variance}$) of potential productivity values (*Ypot*) in our landscape again represent landscape characteristics, which are constant within model runs. We used $Ypot_{mean}$ and $Ypot_{variance}$ as input to establish a beta-distribution. $Ypot_{mean}$ ranges naturally, as the mean of a beta distribution, from 0 to 1. For each $Ypot_{mean}$ value, we then compute the maximal variance that mathematically still allows to establish a beta-distribution ($Ypot_{varmax}$) following the equation

$$Ypot_{varmax} = Ypot_{mean}\,(1 - Ypot_{mean}) \qquad (14)$$

$Ypot_{variance}$ is then calculated as normalisation of the variance used in the beta-distribution by dividing it by $Ypot_{varmax}$. Hence, also $Ypot_{variance}$ ranges between 0 and 1. After setting $Ypot_{mean}$ and $Ypot_{variance}$, we established $Ypot_{mean}$ for the land that is used for agriculture based on drawing *s* values for $\boldsymbol{z_h}$ from the parameterised beta-distribution. *s*, which was set to 10 000 across all simulations, represents thereby the number of equally-sized spatial units (i.e. the number of gird-cells) in a landscape and $\boldsymbol{z_h}$ stands for the *Ypot* of a given cell *h*.

If all drawn $\boldsymbol{z_h}$ values are ordered from the largest to the smallest value, then the element ranked at the position *q*, which defines the lowest yield potential of all agriculturally used cells ($Y_{pot}^{threshold}$) is calculated as

$$q = WL\,(s + 1) \qquad (15)$$

The Y_{pot} of *q* (the value drawn from the beta distribution for the element q) is then equivalent with $Y_{pot}^{threshold}$ and $f_{WL}^{Y}(WL)$ calculated as

$$f_{WL}^{Y}(WL) = \frac{\sum_{h=1,}^{s} z_h\ \mathbf{1}\left(z_h \geq Y_{pot}^{threshold}\right)}{q} \qquad (16)$$

Hence, the function $f_{WL}^{Y}(WL)$ in equation (2) amounts to taking the mean of all random draws from the Beta distribution that are larger or equal to working land.

A2 Model parametrisation

T The three main components of our study were (i) the assessment of biodiversity-production relationships in artificial landscapes, (ii) the contextualisation of model results in archetypic case-studies and (iii) a systematic sensitivity analysis to assess underlying mechanisms triggering intensification traps. In the following section, we describe model parametrisation for each of these three components.

Creation and assessment of artificial landscapes

The goal of the assessment of artificial landscapes was to evaluate biodiversity-production relationships in a wide range of conditions and evaluate the impact of variability in landscape characteristics on the risk of intensification traps. Our primary targets in the process of parametrisation were to (i) attain a large number of datasets for the parametrisation of the eight model constants that we focused on, and (ii) ensure that the applied datasets cover a wide range of different crop types and geographic regions.

Our literature search started with the establishment of a list of search terms for each of the key relationships that were parametrised (listed in Supplementary Table 3). These terms were used as search terms applied to search within title, abstract, keywords and keywords plus of articles in Web of Science. All searches were implemented within the period of 01.12.2022 to 20.03.2023. It was not feasible to implement a full meta-analysis for each model parameter screening all available scientific articles as each search term combination resulted in over thousand returns. Instead, we screened the first hundred returns (sorted by 'relevance setting') of each query for potential data sets that filled the requirements for model constant parametrisation (see below for criteria for individual model constants). This search resulted in data that was often biased covering either a limited range of crops (e.g. wheat, rice or maize) or a limited geographic distribution (primarily originating from industrialised countries). If necessary, we, therefore, complemented our data for parametrisation by using snowball searches starting from key references and by using more region or crop type-targeted search terms to attain data from otherwise underrepresented systems. All references used for parametrisation and the resulting values for individual model constants are listed in Supplementary Data.

Screened articles were filtered based on the following criteria:

Relationship A was parametrised by screening literature for available datasets that provided multiple yield measurements within an otherwise largely homogenous landscape. We, for example, discarded datasets that contained differences in fertiliser or pesticide applications or that showed large differences in the amount of adjacent natural habitat, which could induce biodiversity-driven yield differences. As we controlled for these factors, the variability of yields reflected largely the variation in the agricultural production potential within a landscape. From each of the resulting datasets, mean and standard deviation were computed, which was used to parametrise $Ypot_{mean}$ and $Ypot_{variance}$.

Relationships B, C and D where each defined by two parameters, a slope and an effect size parameter (equations 5-8). The slope parameter was determined using datasets that covered a '*sufficient range'* of the predictor and contained at least 5 different predictor values. In order that a range was considered as sufficiently wide, one of two requirements needed to be fulfilled. Either values ranged from very low values (almost 0) to values that represent the upper range found in natural agroecological landscapes (e.g., in terms of fertiliser applications, etc.). Alternatively, an upper data range was considered as sufficient if it included the saturating section of a non-linear relationship (e.g., in biodiversity – ecosystem function relationships). Once a data-set was considered as suitable for the parametrisation of the slope parameter, we create a fitting grid containing 20301 combinations of parameter values for the shape parameter (ranging from -1 to 1, Supplementary Figure 1) and the effect size parameter (ranging from 0 to 1; both shape and effect size parameters were systematically varied across ranges in steps of 0.01 to create the fitting gird). We then computed for each combination of shape and effect size parameter predictions for the data

points in the parametrisation data set and chose the parameter settings that resulted in the lowest deviations between predictions and real values (i.e. lowest sum of square of residuals). This procedure was repeated for each of the available parametrisation data sets (at least 11 per shape parameter of the key relationships B, C and D).

The effect size parameter can be derived from the change of the response variable across a sufficient range of the predictor variable. In that regard, the same principal requirements for the required data range as for the range of slopes were applied, but only two data points were required (upper and lower end of predictor range). A challenge was that the available datasets for the parametrisation of $Impact_{YI}$, which represents to joint impact of pesticides and fertilisers, are either quantifying the effect of pesticides or fertilisers. Hence, we assessed the impact of pesticides (number of data sets: 11) and fertilisers (number of data sets: 17) separately and combined the two effects by multiplying the results.

$Spill_{IE}$, which is required for the parametrisation of relationship B was estimated as 0.9 reflecting that pesticide and other agricultural practices have a spillover effect on natural communities, which is, however, only of moderate importance (Szabo *et al.* 2012; McArt *et al.* 2017). The variability of $Spill_{IE}$ across landscapes was set to a standard deviation of 0.1.

The parametrisation of relationship E required to establish the regional species pool and hence, setting habitat requirements and a threshold for persistence, H_T, for each species. We created the regional species pool by setting the total number of species to 10,000, which was high enough to avoid stochastic effects having a large impact on model outputs. Further, the average H_T across all species was set to 0.25 after Martin et al. (2019) and we determined H_T values for individual species by drawing from them from a normal distribution with a standard deviation of 0.4. Non-realistic values of below 0 above 1 for individual species were discarded. Also, the habitat requirement of species, i.e. the habitat that is required to persist, was determined stochastically for each individual species. The expected fraction of species that require either *WL*, or *non-WL* or can reproduce in both habitat types was set to values of 0.6, 0.25 and 0.15, respectively and varied stochastically as depicted in Supplementary Figure 3.

Although, the establishment of relationship E is rooted in ecological theory, it represents a highly simplified and conceptualised representation of natural systems. Simplification and conceptualisation help to understand mechanistic dependencies in agroecological systems, but made a parametrisation from real-word data difficult. We nonetheless varied the mean H_T, its standard deviation and the fraction of different habitat requirements in different model runs to represent the variability in natural systems. Yet, the boundaries of their range were chosen arbitrarily (Supplementary Figure 3). Further, we set the variability of mean H_T and the variability of its standard deviation to 0.2 and 0.3, respectively.

Consequently, we established for each model constant a mean value and a standard deviation. These mean values and standard deviation were primarily derived from literature values and if this was not possible, they were carefully chosen to reflect variability in real world situations. Based on mean and standard deviation of real-world data, we established distributions assuming normality. Hence, we were able to create via a bootstrap algorithm artificial landscapes, by drawing for each model constant a value from its distribution.

In real-world situations, shape and effect size of the five key relationships in our conceptual framework are influenced by a combination of environmental (e.g. soil chemistry, climate), biotic (e.g. composition of species pools) and crop (e.g. dependency on pollination and natural

pest control, crop rotation, etc.) characteristics. Hence, one artificial landscape with one set of model constants reflects one set of environmental conditions paired with a specific natural community configuration and a specific crop cultivation scheme. In our analysis, we established 10,000 different artificial landscapes and analysed the associated risk of intensification traps and trade-offs between biodiversity and production. As the model constant that drive differences between these landscapes, were derived based on a broad and extensive literature analysis of real-world data, we presumed that our analytical procedure captures the variability of crop, soil and biotic landscape characteristics encountered across different biomes and geographic regions.

Selection and model parametrisation of archetypal case-studies

The three archetypal case-studies, the US wheat-belt scenario, the South-East Asian rice scenario and the African small-holder scenario, were selected to represent production systems with largely diverging crop, soil and biotic characteristics from different geographic regions.

One major element that differed across archetypal case-studies was crop diversity. In case of the US wheat-belt scenario, a highly industrialised production system based on a wheat monoculture with little crop rotation was chosen, which is commonly encountered in the Great Plains and Northern US (Bushong *et al.* 2012; Vocke & Ali 2013). Similarly, also the South-East Asian rice scenario was predominantly based on one main crop, although an upland rice system was chosen that shows greater degrees of crop rotation (Atlin *et al.* 2006; Saito *et al.* 2018). In contrast, the African small-holder scenario was conceptualised as diversified agro-pastoralist system, a common production form in humid and sub-humid areas of Sub-Saharan Africa (Thornton & Herrero 2015). In these systems, both crop rotation and intercropping are frequently common practice (Conelly & Chaiken 2000; Isaacs *et al.* 2016) with often five or more crop types planted in a single field (A. Burian, personal observation; Supplementary Figure 4). The level of diversification can be even further enhanced by planting timber and fruit trees (Kuyah *et al.* 2019).

Archetypal case-studies represent an idealised form of sometimes quite variable production systems (e.g. the US wheat belt stretches over more than thousand km and covers largely different crop varieties with different features; Vocke & Ali 2013). Additionally, parametrisation of individual case-studies with case-study specific data sets was not possible due to data limitations (some of the measurements necessary for parameterisation show a high degree of stochasticity and hence parameterisation form one or a very small number of literature data is not to a robust practice). We therefore chose model constants for archetypal case-studies using the mean and range of literature data (Supplementary Table 2) as yardstick and qualitative literature evaluation (see below) as reference.

The US wheat-belt scenario was set to have the highest average yield potential and lowest variability of yield potential whereas the African small-holder scenario had the lowest average yield potential and the highest variability of yield potential (note: the yield potential is always normalised to a certain landscape and differences in average yield potential do not denote difference in absolute fertility). These parameters were chosen to reflect the impact of land-use history during past decades, which in the Great Plains through the use of large machinery can drive soil homogenisation (Stover & Henry 2018). In contrast, small-scale farms can be very patchy in e.g. soil qualities and many Sub-Saharan agricultural landscapes show

fragmentary degradation due to non-sustainable land-use practices (Siedenburg 2006; Oostendrop & Zaal 2012).

The effect size of conventional intensification on yields was highest for the South-East Asian rice scenario as rice fields have commonly higher N, P and especially K input requirements than wheat fields (Ludemann *et al.* 2022, considering global crop field areas). The respective effect size was slightly lower for the African small-holder scenario (Supplementary Table 4, Extended Data Fig. 1) as crop types commonly include one or even multiple Fabaceae (see Supplementary Figure 4), reducing required fertiliser inputs.

The effect size of biodiversity on yields was set to comparatively lower values for the non-pollination dependent wheat and rice production systems than for the diversified African small-holder scenario. However, rice has a higher vulnerability to insect predators (Deutsch *et al.* 2018) and natural pest control plays frequently an important role in rice paddy systems (Dominik *et al.* 2017; Zou *et al.* 2020). Hence, the effect size of biodiversity on yields was set higher for the South-East Asian than for US wheat-belt scenario. The African small-holder scenario was reliant on a diverse mix of crops which included also crops with intermediate to large pollination dependency (e.g. mangos, cashew, beans, pumpkin, melon; Klein *et al.* 2007; Bishop & Nakagawa 2021), justifying its higher effect size of biodiversity on yields.

Finally, the effect size of conventional intensification on biodiversity was set highest in the African small-holder scenario and lower in the other two case-studies (Supplementary Table 4). A global assessment of land-use history has shown that historic exposure to disturbance through e.g. agricultural land use results in a higher resistance of communities to land use impacts (Betts *et al.* 2019). Consequently, the longer land use history in the US and South-East Asian case studies compared to often very recent crop-land extensions in Sub-Saharan Africa (Maitima *et al.* 2009; Rudel 2013; Montfort *et al.* 2021) was the justification for this choice. We, nonetheless, acknowledge that the parameters defining the sensitivity of natural systems have been started to be explored (Filgueiras *et al.* 2021), but are only rudimentary understood. The choices for other model constants are displayed in Supplementary Table 4 and the resulting key relationships are presented in Extended Data Fig. 1.

Model parametrisation in sensitivity analysis

The third element of our assessments was the implementation of a systematic sensitivity analysis to evaluate the mechansism underlying the emergence of intensififcation traps. This senstivity analysis targeted each of the five key relationships defined in our conceptual framework (Fig. 1). Relationships A-D had always 2 model constants that together define the shape and effect size of the respective relationship. In a fully factorial design, each of the two variables was changed in steps of 0.01 units between the ranges stated in Supplementary Table 2. All other model constants were maintained at mean literature values (Supplementary Table 2; fractions of species' habitat requirements in the regional species pool were 0.3 for agricultural land, 0.6 for natural habitat and 0.1 for species that could reproduce in both habitat types). For each combination of the two model constants that define one key relationship, a full model run was implemented (i.e. model outputs were computed for all combinations of model inputs). Hence, we could evaluate the response in biodiversity and production in agricultural landscapes to changes in the key relationships defined in our framework.

The senesitivity analysis for relationship E was implemented by altering the expected fractions of species' habitat requirements in the regional species pool. Possible ranges

included 0 - 0.8 for species reproducing only in agricultural land and species that can reproduce in both habitat types. The range for species that require natural habitats for reproduction varied from 0.2 – 1. Habitat requirements were altered in steps of 0.01 units and all possible combinations within the defined ranges of habitat requirement classes were evaluated by implementing a full model run (i.e. model outputs were computed for all combinations of model inputs).

A3 Simplifications in model construction

Naturally, conceptual modelling approaches represent a simplification of natural environments. We highlight in this section the major simplification made in our approach and potential ramifications. Specifically, we will address (i) issues of scale and landscape configuration, (ii) the simplified integration of biodiversity and (iii) the conceptualisation of agricultural land-use practices and (iv) the lack of interaction effects and temporal dynamics in our framework.

(i) *Issues of scale and landscape configuration*: Our framework is designed for the landscape scale, which is variable in extent and is extensively discussed in literature (Fahrig 2005). We restrain from a specification of a fixed grid-size as the appropriate analytical scale is likely case-study specific and e.g. dependant on landscape heterogeneity. Our aim was not to make explicit predictions but to improve our conceptual understanding of intensification traps. Hence, we chose a generic landscape perspective that can be flexibility applied.
Further, our framework accounts for spatial relationships (e.g. ratios of land-use types), but does not integrates explicit spatial landscape configuration. We fully acknowledge that the spatial structure of landscapes is key in mediating both positive and negative spill-over effects from and to natural habitats (Rand, Tylianakis & Tscharntke 2006; Szabo *et al.* 2012; Albrecht *et al.* 2020; Garibaldi *et al.* 2021). However, an integration of explicit landscape structure would have exponentially increased model complexity and made a conceptual synthesis of the drivers of intensification traps very difficult.
Moreover, we normalised biodiversity and agricultural crop production within each analysed landscape. This normalisation facilitats direct comparison across different individual landscapes, but prevents assessments along e.g. absolute productivity or biodiversity gradients, which were not the target of our analyses.

(ii) *Simplifications required to incorporate biodiversity*: We relied on species richness as our metric to capture biodiversity and it was derived based on the requirements of the species present in the regional species pool. However, also other biodiversity metrics such as evenness or genetic diversity can play an important role in regulating agricultural production (Graves, Pearson & Turner 2017; Tarifa *et al.* 2021; Burian *et al.* 2023). Additionally, also the abundance of individual population play a key role in determining their impact on agricultural crops (Dainese *et al.* 2019).
Moreover, biodiversity and other indicators of natural communities have not only positive, but sometimes also negative consequences for crop production (e.g. a high natural biodiversity can also lead to a higher abundance and diversity of pests and diseases). The integration of theses complex interrelationships, i.e. between species richness, evenness and abundance, would have substantially complicated our model structure. Hence, we decided to use species richness as a simple indicator of the impact of natural communities on agricultural production.
Further, there was also an implication of the lack of an exact landscape structure for the representation of biodiversity. This lack of an explicit spatial landscape structure meant that differences in habitat connectivity among patches of non-working land could not be considered. Hence, our model is based on the assumption that landscape design allows for an

efficient colonisation of the different local habitat patches by species in the regional species pool.

(iii) *Conceptualisation of agricultural land-use*: We highlighted and explained already before, why we did only consider conventional intensification and did not explicitly model agroecological practices in our framework. Moreover, conventional intensification was primarily considered as the degree of fertiliser and pesticide use. We readily acknowledge that conventional intensification also incorporates also many other practices, such as irrigation, changes in frequency of tillage and simplification of crop rotations. Yet, these elements of conventional intensification have a much more context-dependent impact on biodiversity and we consequently made the decision to focus on fertilisation and pesticide use as main elements and indicators of conventional intensification (Beckmann *et al.* 2019).

(iv) *Absence of interaction effects and temporal dynamics*: A further important simplification in our framework is the decision to neither consider potential interaction effect among the five key relationships nor specific temporal dynamics. Interaction effects between different drivers of yields can substantially affect the outcome of our model. These interaction effects can both increase or decrease the likelihood of intensification traps. For example, Chen et al. (2022) have recently shown positive interactions between pollination and nutrient fertilisation. In the context of our model, this would imply that a higher level of conventional intensification would have a positive impact on the effect size of biodiversity on yield.
Similar interaction effects have already been reported earlier (Klein *et al.* 2015; Villa-Galaviz *et al.* 2021), but they are not always positive in their nature. For example, a negative interacting effect between pesticide application and natural pest suppression can be expected. I.e., when high levels of pesticides are applied, the benefits generated from natural predators regulating weed or insect pest abundances are decreased. Under such conditions, conventional intensification would have a negative impact on the effect size of biodiversity on yield. Hence, these interaction effects are highly context-dependent. We clearly want to acknowledge here that these effects have the potential to alter model results substantially, but we also argue that adding this high level of complexity into our simulations would have resulted in sometimes counter-intuitive results that would have detracted much of the reader's attention to highly context-specific situations.
Finally, we do not consider temporal variability in our model structure. Temporal variability is essential in agricultural production as a large degree of interannual variability is induced through year-to-year variation in climate but also in other factors such as pest and disease prevalence. Such variability and the resulting temporal dynamics have already been shown to be of high importance in the context of poverty traps (Barrett & Swallow 2006; Barrett & Santos 2014) and can be of equal relevance for intensification traps. However, in this model, we considered the static drivers of intensification traps as the first step to increase our understanding of intensification traps before an added complexity of temporal dynamics is considered.

A4: Additional tables and figures

Supplementary Table 1: Model constant that characterise the artificial landscape that is defined per model run (systematic exploration of all possible land-use combinations).

Landscape characteristics	Limits	Explanation
$Shape_{BI}$	$-1 \leq x \leq 1$	Impact independent shape of the relationship between B_T and I_E: -1 one is a strongly concave and +1 a strongly convex nonlinear relationship
$Impact_{BI}$	$0 \leq x \leq 1$	Maximum impact size of I_E on B_T
$Ypot_{mean}$	$0 \leq x \leq 1$	Mean of potential maximum Y across all areas in a landscape
$Ypot_{variance}$	$0 \leq x \leq 1$	Variance of potential maximum Y across all areas in a landscape
$Shape_{YB}$	$-1 \leq x \leq 1$	Impact independent shape of the relationship between Y and B_T: -1 one is a strongly concave and +1 a strongly convex nonlinear relationship
$Impact_{YB}$	$0 \leq x \leq 1$	Maximum impact size of B_T on Y
$Shape_{YI}$	$0 \leq x \leq 1$	Impact independent shape of the relationship between Y and I_E: -1 one is a strongly concave and +1 a strongly convex nonlinear relationship
$Impact_{YI}$	$0 \leq x \leq 1$	Maximum impact size of I_E on Y
$H_{T.mean}$	$0 \leq x \leq 1$	Threshold value of existence, representing minimum habitat requirements of a species to persist
$H_{T.sd}$	$0 \leq x$	Standard deviation of the threshold value of existence
H_{WL}	$0 \leq x \leq 100$	Percentage of species in the species pool that only live in *WL*
$H_{non\text{-}WL}$	$0 \leq x \leq 100$	Percentage of species in the species pool that live only in *non-WL*
H_{both}	$0 \leq x \leq 100$	Percentage of species that can live in both *WL* and *non-WL*
$Spill_{IE}$	$0 \leq x \leq 2$	Spill-over effects of I_E in working land on $B_{non\text{-}WL}$

Supplementary Table 2: Parametrised model constant for analyses of artificial landscapes and the sensitivity analysis. Stated are the number of datasets used (*n*), the mean and standard deviation of extracted values. Further, we state the lower and upper range of model constants in the systematic sensitivity analysis. *: 17 datasets for fertilisation and 11 for pesticide use.

Relationship in Fig. 1	Model constant	Literature data			Sensitivity analysis	
		n	mean	sd	Lower range	Upper range
A	$Ypot_{mean}$	21	0.45	0.13	0.1	0.9
A	$Ypot_{variance}$	21	0.22	0.13	0.01	0.99
B	$Shape_{YI}$	11	-0.53	0.14	0.2	0.99
B	$Impact_{YI}$	27*	0.77	0.28	-1	0
C	$Shape_{YB}$	17	-0.36	0.40	0.01	0.99
C	$Impact_{YB}$	26	0.60	0.24	-1	1
D	$Shape_{BI}$	14	0.25	0.37	0.2	0.99
D	$Impact_{BI}$	21	0.67	0.17	-1	1

Supplementary Table 3: Searched terms used for the literature queries to gather dataset for the parametrisation of key relationships A-D.

Key relationship	Model constants	Search terms
A	$Ypot_{mean}$, $Ypot_{variance}$	("production" OR "productivity") AND ("crop" OR "agricultur*" OR "fruit" OR "hay") AND ("mean" OR "variability" OR "standard deviation" OR "variance")
B	$Shape_{YI}$, $Impact_{YI}$	("production" OR "productivity" OR "Yield") AND ("crop" OR "agricultur*" OR "fruit" OR "hay" OR "fruit") AND ("fertiliser" OR "intensity" OR "intensification" OR "pesticide")
C	$Shape_{YB}$, $Impact_{YB}$	("biodiversity" OR "richness" OR "phylogenetic diversity") AND ((("functionality" OR "function") AND ("agriculture" OR "soil")) OR "pollination" OR "pest control" OR "fertility" OR "flower visits" OR "seed predation"OR"production"OR"productivity")
D	$Shape_{BI}$, $Impact_{BI}$	("sensitivity" OR "resistance" OR "response") AND ("pesticide" OR "herbicide" OR "fungicide" OR "eutrophication" OR "insecticide") AND ("animal" OR "insect" OR "bird" OR "pollinator" OR "fauna" OR "biodiversity" OR "richness")

Supplementary Table 4: Model constant chosen for the archetypal case-studies in relation to mean literature values.

Relationship in Fig. 1	Model constant	Mean Literature data	Wheat case-study	Rise case-study	SSA small-holder case-study
A	$Ypot_{mean}$	0.45	0.70	0.65	0.40
A	$Ypot_{variance}$	0.22	0.20	0.30	0.35
B	$Shape_{YI}$	-0.53	-0.60	-0.40	-0.30
B	$Impact_{YI}$	0.77	0.85	0.95	0.70
C	$Shape_{YB}$	-0.36	-0.50	-0.40	-0.50
C	$Impact_{YB}$	0.60	0.30	0.60	0.70
D	$Shape_{BI}$	0.25	0.30	0.30	-0.20
D	$Impact_{BI}$	0.67	0.50	0.70	0.85
E	H_{WL}	0.15	0.05	0.30	0.05
E	$H_{non\text{-}WL}$	0.60	0.70	0.65	0.70
E	H_{both}	0.25	0.25	0.05	0.25

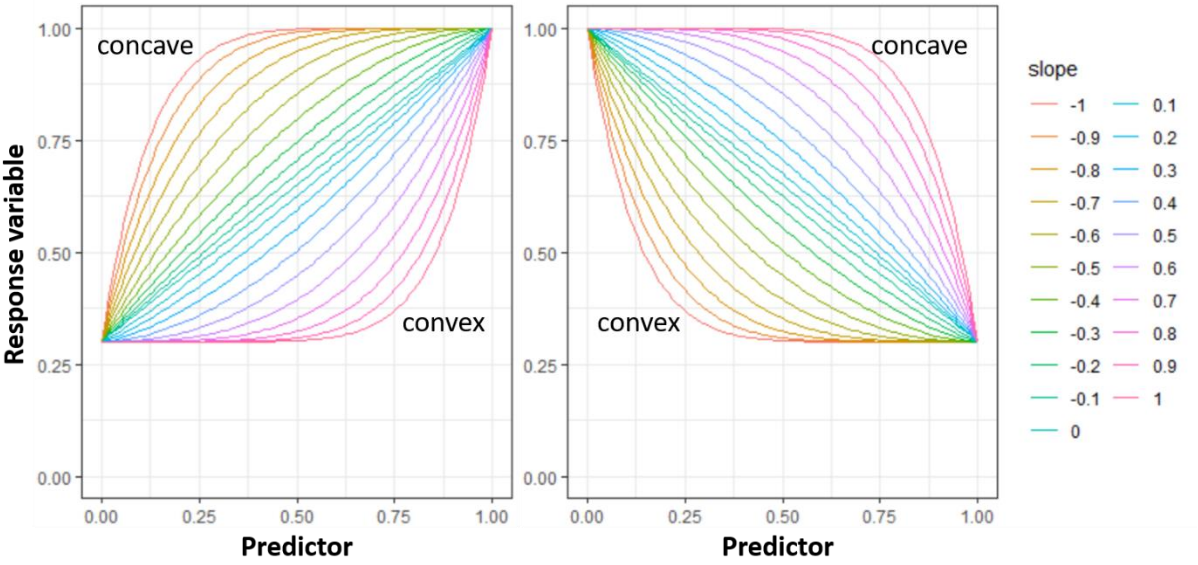

Supplementary Figure 1: Possible shapes of the relationship between predictor and response variable in negative (left) and positive (right) relationships. Extreme endpoints of relationships range between highly concave (shape constant of -1) and highly convex (shape constant of 1) with a shape constant of 0 indicating a linear relationship.

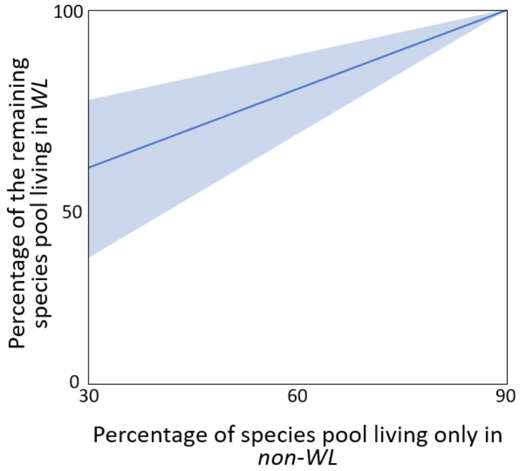

Supplementary Figure 2: In the creation of artificial landscapes, the percentage of species that can survive in *non-WL* ranged between 30 and 90%. 90% would represent a landscape where natural habitat is highly diverse (e.g. tropical rainforest), whilst 30% would indicate that the original natural habitat is relatively poor in species and that transformations into cropland with associated fertilisation can increase species richness (e.g. situation frequently encountered in tropical soils (Barnes *et al.* 2017)). Further, we set the range of species living in *WL* to 10-70% of the total species pool. We thereby defined a minimum level of 10% based on the reasoning that there will be always some species that are specialised to the planted crops and that will not be able to persist in natural habitats. Finally, the resulting range of species that can survive in both *WL* and *non-WL* was 0-60%. The graph displays the impact of changes in the percentage of species living in *non-WL* on the percentage of the remaining species that live in *WL*. The blue line represents most frequent values and the shaded area the range that is described by the 68.2% prediction interval.

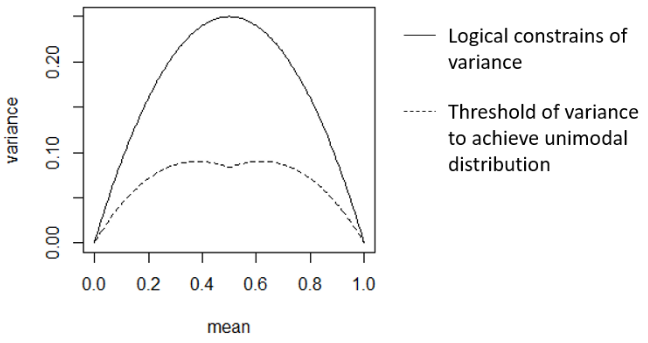

Supplementary Figure 3: Logical constrain of variance (solid line) and the variance threshold to achieve unimodal distributions (dashed line) in beta distributions in relation to different mean values.

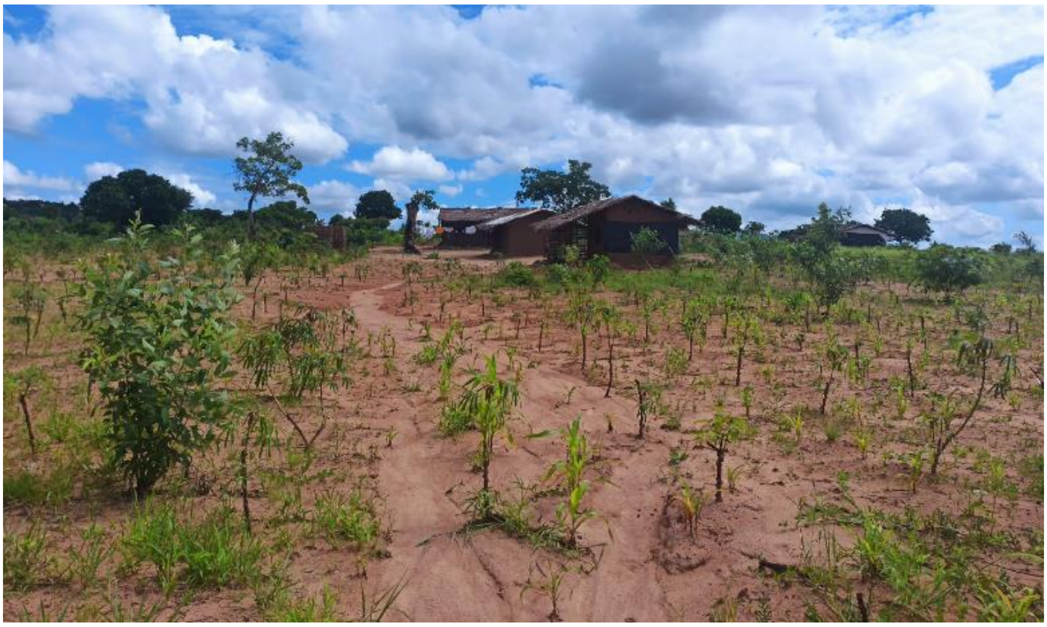

Supplementary Figure 4: Example of African small-holder system in Northern Mozambique. Within a small area, cassava is intercropped with maize, bean, peanuts, sweet potatoes and pumpkins. Additionally, cashew, papaya and mango trees as well as other medicinal and timber trees are used to create an agroforestry system that is high in crop diversity at the field level.

A5: References

Albrecht, M., Kleijn, D., Williams, N.M., Tschumi, M., Blaauw, B.R., Bommarco, R., Campbell, A.J., Dainese, M., Drummond, F.A. & Entling, M.H. (2020) The effectiveness of flower strips and hedgerows on pest control, pollination services and crop yield: a quantitative synthesis. *Ecology letters,* **23,** 1488-1498.

Atlin, G., Lafitte, H., Tao, D., Laza, M., Amante, M. & Courtois, B. (2006) Developing rice cultivars for high-fertility upland systems in the Asian tropics. *Field crops research,* **97,** 43-52.

Barnes, A.D., Allen, K., Kreft, H., Corre, M.D., Jochum, M., Veldkamp, E., Clough, Y., Daniel, R., Darras, K. & Denmead, L.H. (2017) Direct and cascading impacts of tropical land-use change on multi-trophic biodiversity. *Nature Ecology & Evolution,* **1,** 1511-1519.

Barrett, C.B. & Santos, P. (2014) The impact of changing rainfall variability on resource-dependent wealth dynamics. *Ecological Economics,* **105,** 48-54.

Barrett, C.B. & Swallow, B.M. (2006) Fractal poverty traps. *World Development,* **34,** 1-15.

Beckmann, M., Gerstner, K., Akin-Fajiye, M., Ceaușu, S., Kambach, S., Kinlock, N.L., Phillips, H.R., Verhagen, W., Gurevitch, J. & Klotz, S. (2019) Conventional land-use intensification reduces species richness and increases production: A global meta-analysis. *Global Change Biology,* **25,** 1941-1956.

Betts, M.G., Wolf, C., Pfeifer, M., Banks-Leite, C., Arroyo-Rodríguez, V., Ribeiro, D.B., Barlow, J., Eigenbrod, F., Faria, D. & Fletcher Jr, R.J. (2019) Extinction filters mediate the global effects of habitat fragmentation on animals. *Science,* **366,** 1236-1239.

Bishop, J. & Nakagawa, S. (2021) Quantifying crop pollinator dependence and its heterogeneity using multi-level meta-analysis. *Journal of Applied Ecology,* **58,** 1030-1042.

Brühl, C.A., Bakanov, N., Köthe, S., Eichler, L., Sorg, M., Hörren, T., Mühlethaler, R., Meinel, G. & Lehmann, G.U. (2021) Direct pesticide exposure of insects in nature conservation areas in Germany. *Scientific Reports,* **11,** 1-10.

Burian, A., Norton, B.A., Alston, D., Willmot, A., Reynolds, S., Meynell, G., Lynch, P. & Bulling, M. (2023) Low-cost management interventions and their impact on multilevel trade-offs in agricultural grasslands. *Journal of Applied Ecology*.

Bushong, J.A., Griffith, A.P., Peeper, T.F. & Epplin, F.M. (2012) Continuous winter wheat versus a winter canola–winter wheat rotation. *Agronomy Journal,* **104,** 324-330.

Chen, K., Kleijn, D., Scheper, J. & Fijen, T.P. (2022) Additive and synergistic effects of arbuscular mycorrhizal fungi, insect pollination and nutrient availability in a perennial fruit crop. *Agriculture, Ecosystems & Environment,* **325,** 107742.

Conelly, W.T. & Chaiken, M.S. (2000) Intensive farming, agro-diversity, and food security under conditions of extreme population pressure in Western Kenya. *Human Ecology,* **28,** 19-51.

Dainese, M., Martin, E.A., Aizen, M.A., Albrecht, M., Bartomeus, I., Bommarco, R., Carvalheiro, L.G., Chaplin-Kramer, R., Gagic, V. & Garibaldi, L.A. (2019) A global synthesis reveals biodiversity-mediated benefits for crop production. *Science advances,* **5,** eaax0121.

Deutsch, C.A., Tewksbury, J.J., Tigchelaar, M., Battisti, D.S., Merrill, S.C., Huey, R.B. & Naylor, R.L. (2018) Increase in crop losses to insect pests in a warming climate. *Science,* **361,** 916-919.

Dominik, C., Seppelt, R., Horgan, F.G., Marquez, L., Settele, J. & Václavík, T. (2017) Regional-scale effects override the influence of fine-scale landscape heterogeneity on rice arthropod communities. *Agriculture, Ecosystems & Environment,* **246,** 269-278.

Fahrig, L. (2005) When is a landscape perspective important. *Issues and perspectives in landscape ecology***,** 3-10.

Filgueiras, B.K., Peres, C.A., Melo, F.P., Leal, I.R. & Tabarelli, M. (2021) Winner–loser species replacements in human-modified landscapes. *Trends in Ecology & Evolution,* **36,** 545-555.

Garibaldi, L.A., Oddi, F.J., Miguez, F.E., Bartomeus, I., Orr, M.C., Jobbágy, E.G., Kremen, C., Schulte, L.A., Hughes, A.C. & Bagnato, C. (2021) Working landscapes need at least 20% native habitat. *Conservation Letters,* **14,** e12773.

Graves, R.A., Pearson, S.M. & Turner, M.G. (2017) Species richness alone does not predict cultural ecosystem service value. *Proceedings of the National Academy of Sciences,* **114,** 3774-3779.

Isaacs, K.B., Snapp, S.S., Chung, K. & Waldman, K.B. (2016) Assessing the value of diverse cropping systems under a new agricultural policy environment in Rwanda. *Food Security,* **8,** 491-506.

Klein, A.-M., Vaissiere, B.E., Cane, J.H., Steffan-Dewenter, I., Cunningham, S.A., Kremen, C. & Tscharntke, T. (2007) Importance of pollinators in changing landscapes for world crops. *Proceedings of the Royal Society B: Biological Sciences,* **274,** 303-313.

Klein, A.M., Hendrix, S., Clough, Y., Scofield, A. & Kremen, C. (2015) Interacting effects of pollination, water and nutrients on fruit tree performance. *Plant biology,* **17,** 201-208.

Kraft, N.J., Adler, P.B., Godoy, O., James, E.C., Fuller, S. & Levine, J.M. (2015) Community assembly, coexistence and the environmental filtering metaphor. *Functional ecology,* **29,** 592-599.

Kuyah, S., Whitney, C.W., Jonsson, M., Sileshi, G.W., Öborn, I., Muthuri, C.W. & Luedeling, E. (2019) Agroforestry delivers a win-win solution for ecosystem services in sub-Saharan Africa. A meta-analysis. pp. 1-18. Springer.

Liu, W., Yang, H., Folberth, C., Müller, C., Ciais, P., Abbaspour, K.C. & Schulin, R. (2018) Achieving high crop yields with low nitrogen emissions in global agricultural input intensification. *Environmental science & technology,* **52,** 13782-13791.

Ludemann, C.I., Gruere, A., Heffer, P. & Dobermann, A. (2022) Global data on fertilizer use by crop and by country. *Scientific data,* **9,** 501.

Maitima, J.M., Mugatha, S.M., Reid, R.S., Gachimbi, L.N., Majule, A., Lyaruu, H., Pomery, D., Mathai, S. & Mugisha, S. (2009) The linkages between land use change, land degradation and biodiversity across East Africa. *African Journal of Environmental Science and Technology,* **3**.

Martin, E.A., Dainese, M., Clough, Y., Báldi, A., Bommarco, R., Gagic, V., Garratt, M.P., Holzschuh, A., Kleijn, D. & Kovács-Hostyánszki, A. (2019) The interplay of landscape composition and configuration: new pathways to manage functional biodiversity and agroecosystem services across Europe. *Ecology letters,* **22,** 1083-1094.

McArt, S.H., Urbanowicz, C., McCoshum, S., Irwin, R.E. & Adler, L.S. (2017) Landscape predictors of pathogen prevalence and range contractions in US bumblebees. *Proceedings of the Royal Society B: Biological Sciences,* **284,** 20172181.

Montfort, F., Begue, A., Leroux, L., Blanc, L., Gond, V., Cambule, A.H., Remane, I.A. & Grinand, C. (2021) From land productivity trends to land degradation assessment in Mozambique: Effects of climate, human activities and stakeholder definitions. *Land Degradation & Development,* **32,** 49-65.

Oostendrop, R. & Zaal, F. (2012) Farm- and household-level drivers of agricultural innovation in machakos and Kitui districts, Kenya. *Sustainable land management in the tropics: Explaining the miracle* (eds F. Zaal & K. Burger), pp. 45-64. Ashagte Publishing, Farnham.

Pywell, R.F., Heard, M.S., Woodcock, B.A., Hinsley, S., Ridding, L., Nowakowski, M. & Bullock, J.M. (2015) Wildlife-friendly farming increases crop yield: evidence for ecological intensification. *Proceedings of the Royal Society B: Biological Sciences,* **282,** 20151740.

Rand, T.A., Tylianakis, J.M. & Tscharntke, T. (2006) Spillover edge effects: the dispersal of agriculturally subsidized insect natural enemies into adjacent natural habitats. *Ecology letters,* **9,** 603-614.

Rudel, T.K. (2013) The national determinants of deforestation in sub-Saharan Africa. *Philosophical Transactions of the Royal Society B: Biological Sciences,* **368,** 20120405.

Saito, K., Asai, H., Zhao, D., Laborte, A.G. & Grenier, C. (2018) Progress in varietal improvement for increasing upland rice productivity in the tropics. *Plant Production Science,* **21,** 145-158.

Scaife, M. (1968) Maize fertilizer experiments in Western Tanzania. *The Journal of Agricultural Science,* **70,** 209-222.

Siedenburg, J. (2006) The Machakos case study: Solid outcomes, unhelpful hyperbole. *Development Policy Review,* **24,** 75-85.

Stover, H.J. & Henry, H.A. (2018) Soil homogenization and microedges: perspectives on soil-based drivers of plant diversity and ecosystem processes. *Ecosphere,* **9,** e02289.

Szabo, N.D., Colla, S.R., Wagner, D.L., Gall, L.F. & Kerr, J.T. (2012) Do pathogen spillover, pesticide use, or habitat loss explain recent North American bumblebee declines? *Conservation Letters,* **5,** 232-239.

Tarifa, R., Martínez-Núñez, C., Valera, F., González-Varo, J.P., Salido, T. & Rey, P.J. (2021) Agricultural intensification erodes taxonomic and functional diversity in Mediterranean olive groves by filtering out rare species. *Journal of Applied Ecology,* **58,** 2266-2276.

Thornton, P.K. & Herrero, M. (2015) Adapting to climate change in the mixed crop and livestock farming systems in sub-Saharan Africa. *Nature Climate Change,* **5,** 830-836.

Villa-Galaviz, E., Smart, S.M., Clare, E.L., Ward, S.E. & Memmott, J. (2021) Differential effects of fertilisers on pollination and parasitoid interaction networks. *Journal of Animal Ecology,* **90,** 404-414.

Vocke, G. & Ali, M. (2013) US wheat production practices, costs, and yields: Variations across regions.

Zou, Y., De Kraker, J., Bianchi, F.J., Xiao, H., Huang, J., Deng, X., Hou, L. & Van Der Werf, W. (2020) Do diverse landscapes provide for effective natural pest control in subtropical rice? *Journal of Applied Ecology,* **57,** 170-180.
